# Does Exist a Differential Impact of Degarelix *Versus* LHRH Agonists on Cardiovascular Safety? Evidences From Randomized and Real-World Studies

**DOI:** 10.3389/fendo.2021.695170

**Published:** 2021-06-14

**Authors:** Alessandro Sciarra, Gian Maria Busetto, Stefano Salciccia, Francesco Del Giudice, Martina Maggi, Felice Crocetto, Matteo Ferro, Ettore De Berardinis, Roberto Mario Scarpa, Francesco Porpiglia, Luca Carmignani, Rocco Damiano, Walter Artibani, Giuseppe Carrieri

**Affiliations:** ^1^ Department of Urology, Sapienza Rome University Policlinico Umberto I, Rome, Italy; ^2^ Department of Urology and Renal Transplantation, University of Foggia, Policlinico Riuniti, Foggia, Italy; ^3^ Department of Neurosciences, Human Reproduction and Odontostomatology, University of Naples Federico II, Naples, Italy; ^4^ Department of Urology, IRCCS European Institute of Oncology (IEO), Milan, Italy; ^5^ Department of Urology, University Campus Biomedico, Rome, Italy; ^6^ Division of Urology, Department of Oncology, School of Medicine, University of Turin, San Luigi Hospital, Turin, Italy; ^7^ Department of Urology, San Donato Policlinic Hospital, Milan, Italy; ^8^ Department of Urology, Magna Graecia University of Catanzaro, Catanzaro, Italy; ^9^ Department of Urology, Abano Terme Policlinic, Abano Terme, Italy

**Keywords:** prostate cancer, degarelix, LHRH agonists/GnRH antagonists, androgen deprivation therapy, cardiovascular safety

## Abstract

The main systemic therapy for the management of hormone-sensitive prostate cancer (PC) is androgen deprivation therapy (ADT), with the use of long-acting luteinizing hormone releasing-hormone (LHRH) agonists considered the main form of ADT used in clinical practice to obtain castration in PC. The concomitant administration of antiandrogens for the first weeks could reduce the incidence of clinical effects related to the testosterone flare-up in the first injection of LHRH. On the contrary, Gonadotropin Rh (GnRH) antagonists produce a rapid decrease of testosterone levels without the initial flare-up, with degarelix commonly used in clinical practice to induce castration in PC patients. Even if no long-term data are reported in terms of survival to define a superiority of GnRH or LHRH, for oncological efficacy and PC control, data from randomized clinical trials and from real-life experiences, suggest a difference in cardiovascular risk of patients starting ADT. The age-related decline in testosterone levels may represent a factor connected to the increase of cardiovascular disease risk, however, the role of ADT in increasing CV events remains controversial. For these reasons, the aim of the paper is to synthesize the difference in cardiovascular risk between LHRH and degarelix in patients undergoing ADT. A difference in cardiovascular risk could be indeed an important parameter in the evaluation of these two forms of castration therapy. The Randomized trials analyzed in this paper sustain a possible protective role for degarelix versus LHRH agonists in reducing the rate of new CV events and interventions in the short-term period. On the contrary, real-word data are contradictory in different national experiences and are strongly conditioned by huge differences between the LHRH agonists group and the degarelix group.

## Introduction

Androgen deprivation therapy (ADT) is considered the main systemic therapy for the management of hormone-sensitive prostate cancer (PC) although new generation hormone therapies have been developed. Historically, testosterone serum levels considered in clinical trials to obtain castration are still < 50 ng/dl. However, different evidence underlined as better results can be obtained with levels lower than 20 ng/dl (1–3).

Long-acting luteinizing hormone releasing-hormone (LHRH) agonists are currently the main form of ADT used in clinical practice to obtain castration in PC. The first injection induces a transient increase (flare-up) in testosterone levels which lasts approximately one week. This testosterone surge, particularly in high-risk patients, may lead to negative clinical effects such as bone pain, bladder outlet obstruction, cardiovascular (CV) complications (4). Concomitant administration of antiandrogen for the first weeks can reduce the incidence of the clinical effects related to the testosterone surge but not completely remove the risk (5).

Gonadotropin RH (GnRH) antagonists produce a rapid decrease in testosterone serum levels without the initial flare-up. Degarelix is the GnRH antagonist used in clinical practice to induce castration in PC patients.

As underlined by the EAU guidelines (5), the lack of significant long-term data beyond 12 months or survival evidence directly comparing degarelix versus LHRH agonists does not consent to sustain a superiority of one compound on the other in terms of oncological efficacy and PC control.

On the contrary, data from randomized clinical trials and more recently from real-life experiences suggest different cardiovascular morbidity associated with agonists versus antagonists, with a protective role of degarelix in reducing the rate of new CV events and interventions. It has been hypothesized that the determination of the cardiovascular risk of patients starting ADT should be a parameter to choose between these two forms of castration therapy. The aim of the study is to summarize recent evidence in literature comparing LHRH and GnRH in cardiovascular risk in patients starting ADT.

## Cardiovascular Morbidity and Testosterone Castration Levels

The age-related decline in testosterone serum levels in men has been described as a possible cause for the increased risk of hypertension and cardiovascular diseases (CVD). Testosterone can activate both vasodilator and vasoconstrictor pathways, but it is more pro-hypertensive in different models (6). Testosterone is also an anabolic hormone promoting muscle mass, fat loss, and therefore low levels of testosterone can be associated with a metabolic syndrome involving obesity and hypertension (7). In some observational studies an inverse correlation between testosterone serum levels and blood pressure or CVD risk has been shown (8, 9). Qu et al. (6) in a population-based, cross-sectional study on 6296 men reported an inverse correlation between testosterone (total, free testosterone, and sex hormone-binding globulin) levels and the prevalence of hypertension or CVDs. Age > 65 years and body mass index > 24 negatively impacted the inverse association between testosterone and hypertension (**Table 1**).

**Table 1:** Comparison between groups in terms of CVD risk.

Comparison between groups	HR/OR (95% CI) in terms of CVD risk
Normal testosterone *versus* low (< 20 ng/dl) testosterone levels	OR 0.79 (0.69-0.90); p=0.026
Prostate cancer cases *versus* general population	OR 9.45 (4.53- 19.73); p=0.012
ADT treatment *versus* no ADT treatment	HR 0.888 (0.808-0.975); p=0.013

Zhang et al. (10) recently demonstrated that cancer survivors have a higher risk of developing or dying from CVD compared to the general population. In particular, on more than 15000 participants and 1600 cancer survivors, specifically those with bladder, kidney, prostate (OR 9.45; 95%CI 4.53- 19.73), colorectal, lung, melanoma, or testicular cancer had a 2.72-10-47 higher odds of elevated 10-year atherosclerotic CVD (ASCVD) risk (**Table 1**).

Sun et al. (11) performed a cross-sectional analysis on 90494 US men with PC, 22700 submitted to ADT. Patients receiving ADT were more likely to be older and more frequently had a history of ASCVD (21.0% versus 15.5%).

Kim et al. (12) in a study cohort of 131189 newly diagnosed PC divided into ADT and non-ADT groups, analyzed the incidence of newly developed CVD and cardiovascular intervention (CVI). Differently to previous evidence, at multivariate analysis this study reported a reduced risk of CVD and CVI in patients using ADT for 2-3 years (HR 0.888; 95%; CI 0.808-0.975; p=0.0131) or more than 3 years (HR 0.860;95% CI 0.804-0.920; p<0.0001) (**Table 1**).

The relationship between ADT and the development of new CVD remains uncertain because of conflicting evidence. Probably other factors such as increased mean age and a higher incidence of preexisting CVD are more relevant than the long-term use of ADT in determining CV toxic effects (13).

## Literature Search

We searched the electronic databases (MEDLINE, Web of Science, Cochrane Library, and Scopus) in the last twenty years for trials analyzing GnRH antagonist and agonist in terms of cardiovascular impact and safety (prostate cancer and cardiovascular events and degarelix or LHRH agonists).

**Figure 1:**
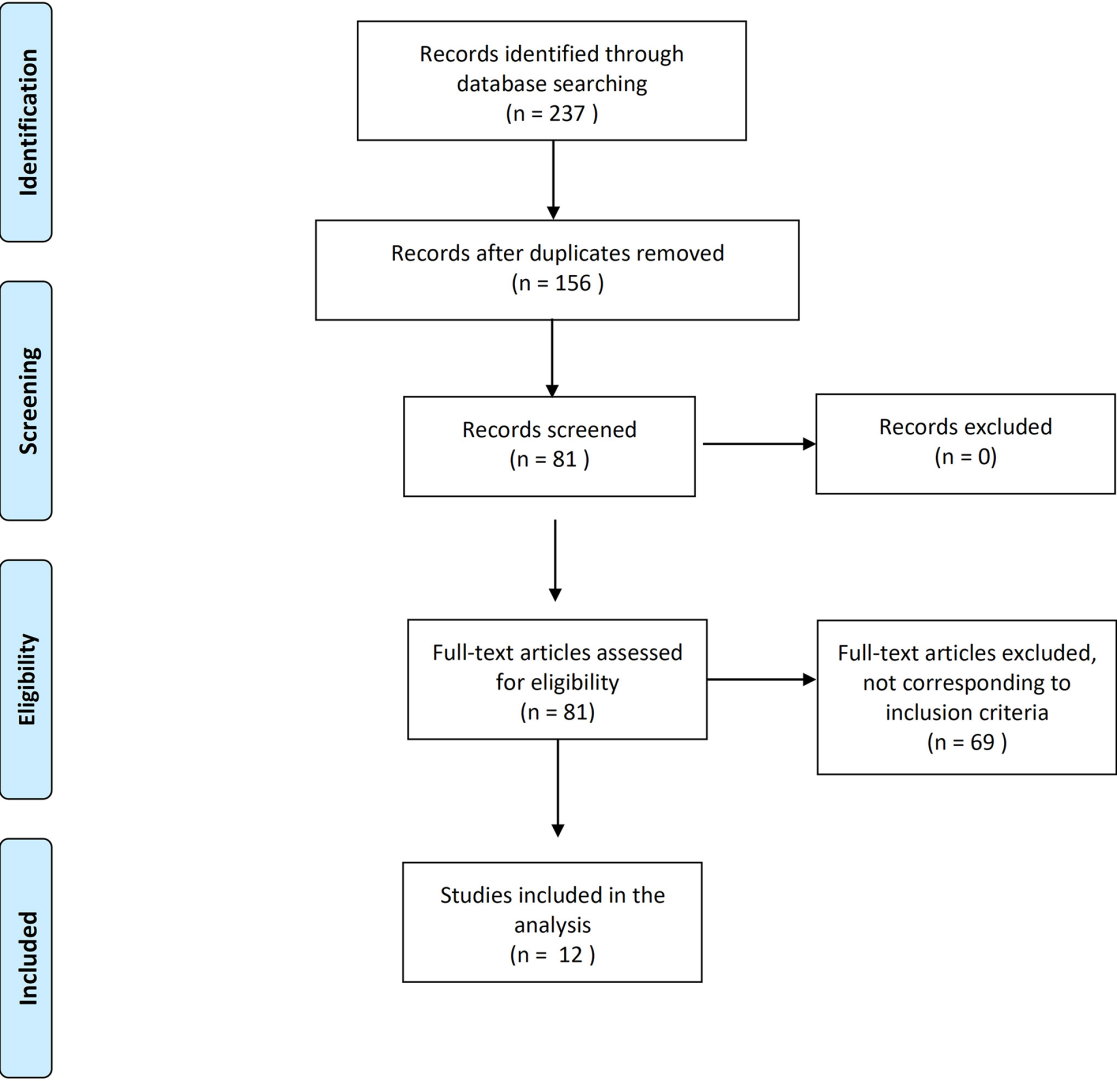
Results of the screening process.

The search was limited to the papers published or in press in peer-reviewed scientific journals or conference proceedings in the last 20 years, published in English and with full-text available. The following inclusion criteria were applied: patients >18years old; patients with locally advanced or metastatic PCA; patient without castration-resistant disease; and at least one of the following outcomes reported (major adverse cardiovascular events – MACE, coronary artery disease – CAD, cerebrovascular accidents – CVA, atrial fibrillation -AF, and heart failure – HF). The result of the screening process is reported in **Figure 1**. The selected studies were classified in prospective comparative analysis between antagonists and agonists (including also meta-analysis) and real-world experiences.

## Impact of LHRH Agonists Versus GnRH Antagonists in Terms of CV Morbidity: Evidence From Randomized Trials

GnRH antagonists reduce testosterone levels without producing the initial flare-up which may cause, analogously, a clinical flare up, triggering a rapid onset of symptoms related to PC (as spinal cord compression or pain related to osteoblastic metastases). Although several antagonists have been investigated, only two of these, degarelix and abarelix, are currently available for clinical use in patients with PC.

Abarelix was associated with rapid decreases in LH and FSH levels in US phase III trials (14, 15), however in Phase III European trial, escape from castration was more common and quicker with abarelix (22%) than with GnRH agonist plus antiandrogen (8%) (p=0.007) (16).

In several Phase III studies, abarelix has been shown to have a safety profile comparable to that of leuprolide or bicalutamide (14–16), but immediate onset of systemic allergic reactions was more commonly observed in a higher number of patients with a cumulative risk, that increased with duration of treatment (17). Unlike degarelix, abarelix therapy has not been thoroughly evaluated in a consistent number of studies. Indeed, to our best knowledge, no significant studies are reported on the comparison between abarelix therapy and LHRH-agonists therapy in terms of CV morbidity. Conversely, degarelix is the most studied third-generation GnRH antagonists, with the advantage of having a rapid and valid testosterone suppression in absence of important allergic reactions due to a reduced histamine-releasing activity which was, instead, associated with previous GnRH antagonists (18).

Eight phase III randomized trials have compared, in the same population of locally advanced and metastatic PC cases, the ADT treatments using degarelix versus LHRH agonists also in terms of CVD (19). In only one trial (20) the determination of CV events rate was the primary endpoint of the study. Margel et al. (20) randomized 80 PC cases (only 26% metastatic) with pre-existing CVDs to 12 months treatment with degarelix versus LHRH agonist. A new CV event developed in 33% and 5% of cases submitted to degarelix and LHRH agonists respectively (p=0.001), with a median time of 8.8 months.

Abufaray et al. (21) conducted a meta-analysis on these 8 randomized trials comprising 2633 patients (1646 treated with degarelix and 986 with LHRH agonists). Populations in all studies included either non-metastatic progressive, locally advanced PC, or metastatic cases. In six out of the eight studies, antiandrogens were shortly associated to agonists to suppress testosterone flare. Degarelix was associated with a lower rate of CV events (RR 0.52; 95% CI 0.34-0.80; p=0.003) with a low heterogeneity in the pooled analysis (I2 = 42%) (**Table 2**). Median Follow-up was only 3 months in three out of the eight studies and 12-14 months in the other; therefore, the analysis of CV events in randomized trials is limited to a short-term period no longer than 12 months.

**Table 2:** Comparison between Degarelix and LHRH agonists in terms of CVD risk.

Comparison between groups	HR/RR (95% CI) in terms of CVD risk
**Degarelix *versus* LHRH agonists in randomized trials (pooled analysis)**	
-Abufaray et al. (21)	RR 0.52; (0.34-0.80); p=0.003) (I^2 =^ 42%)
-Cirne et al. (22)	RR 0.52; (0.28-0.97) (I^2 =^ 21%)
**Degarelix *versus* LHRH agonists in real-world**	
-Davey et al. (23)	HR 0.39 (0.19-0.79); p=0.01
-Perrone et al. (24)	HR 0.76 (0.60-0.95); p=0.018
-George, G (25).	HR 1.25 (0.96-1.61); p> 0.05

Cirne et al. (22) in their recent meta-analysis included also other studies such as the CS35A trial where data are presented only in abstract, and the HERO trial with relugolix was used as GnRH antagonist (Shore NEJM 2020). This meta-analysis was focused on CVD rates underlying that only in three out of ten randomized trials the prevalence of CVD at baseline was described. On 2415 cases submitted to GnRH antagonist 3.4% of CV events were reported whereas on 1345 LHRH agonists 6.5% CV rate was described. The pooled RR for CV events for GnRH antagonists versus agonists was 0.57 (95% CI 0.39-0.81) with low evidence of heterogeneity I2 20.8%). Similar results were obtained considering only trials using degarelix as GnRH antagonist (RR 0.52; 95% CI 0.28-0.97) (**Table 2**).

## Impact of LHRH Agonists *Versus* GnRH Antagonists in Terms of CV Morbidity: Evidence From Real-World Experiences

Recently some real-world data from a national database comparing CV outcomes in PC cases treated with degarelix versus LHRH agonists have been published, while no relevant studies are reported in literature comparing abarelix and LHRH agonists in terms of CV morbidity.

Regarding degarelix, Davey et al. (23) conducted a *post hoc* analysis of real-world data from the UK general practitioner (GP) database (OPCRD) between 2010 and 2017. Over 700 GP collaborate with a population of 9081 PC cases submitted to ADT. The analysis confirmed that most of the cases were submitted to LHRH agonists (8980 cases) when compared to degarelix (101 cases). The follow-up duration of the observation is not reported, and the authors did not specify when and how antiandrogens were associated with LHRH agonists. Authors showed that the relative risk of experiencing any CV event (heart failure, myocardial infarction, arrhythmia) was lower with degarelix than all LHRH agonists (RR 6.9% *versus* 17.7%; 0.39; 95% CI 0.19-0.79; p=0.01) (**Table 2**) and the incidence of heart failure and arrhythmia was particularly lower in cases treated with degarelix.

Perrone et al. (24) presented data from an Italian observational retrospective cohort study based on an administrative database focused on PC cases treated with degarelix *versus* LHRH agonists. In 9785 cases, 93.6% (9158) were treated with agonists and only 6.4% (627) with degarelix. Most of the cases (70%) had at baseline hypertension and cases in the LHRH agonists group were significantly older (mean 76.9 *versus* 74.8 years). At a median follow-up of 33.3 months a higher percentage of cases under LHRH agonists developed CV events (8.8 versus 6.2 per 100 person-years; p=0.002) and at multivariate analysis degarelix treatment was associated with a lower risk for CV events (HR 0.76; 95% CI 0.60-0.95; p=0.018), independently to a previous history of CVD (**Table 2**).

George et al. (26) combined observational data from five European countries to investigate differences in CV events between degarelix and LHRH agonists treatment in locally advanced and metastatic PC. Median Follow-up was 1.8 and 1.2 years for LHRH agonists and degarelix respectively. Also, in this analysis most of the cases were submitted to LHRH agonists (48757 and 2144 PC cases respectively under LHRH agonists and degarelix). Data showed that there was no significant increased risk for developing any CD event in both groups (HR 1.25; 95% CI 0.96-1.61) (**Table 2**). However, cases under degarelix showed a higher risk of developing myocardial infarction (HR 1.62;95% CI; 1.11-2.35) and arrhythmia (HR 1.55; 95% CI 1.11-2.15).

Real-world observational data from 2382 PC cases from a German registry showed no significant differences in the incidence of CVD between LHRH agonists and GnRH antagonists, although a significant increase in hypertension was reported in LHRH agonists (16.4%) compared to cases treated with GnRH antagonist (6.9%; p=0.022) (27).

## Discussion and Critical Analysis

LHRH agonists and GnRH antagonists suppress testosterone levels through different mechanisms and in particular agonists produce an initial testosterone surge with possible clinical effects that can be only in part prevented by the short-time association of an antiandrogen. In patients receiving ADT, this different mechanism to obtain castration may produce different CV risks. The greater extension in FSH levels reduction produced by degarelix may be another mechanism able to reduce the risk for atherosclerosis-related CV events (28). FSH suppression could be potentially important because of its role in the regulation of obesity and FSH hormone receptors on blood vessels (28); however clinical data supporting this relationship are missing.

Randomized trials in which the same population is submitted to degarelix versus LHRH agonists may represent the best setting to analyze possible differences in terms of safety and CV new events. Eight main trials correspond to these requisites, however, all showing some limitations: baseline characteristics in terms of pre-existing CVD are mainly not described, populations include either locally advanced or metastatic PC without stratification of results in terms of tumor stage and follow-up is very limited to 3-12 months. A pooled analysis of the results of these randomized trials sustains that degarelix treatment, in the short-term period of 12 months, is associated with a reduced risk of new CV events when compared to LHRH agonists. During 1 year of treatment, PC cases (mainly non-metastatic) with pre-existing CVDs develop a lower incidence of new CV events using degarelix than LHRH agonists, but this finding is mainly examined in only one study with a limited (80 cases) population.

Real-world observational data could produce relevant information in a real-life situation so to confirm or not a possible relationship between CV events and the use of different ADTs. On the other hand, these observational studies could be vulnerable to bias in terms of patient selection and data collection. The major limit in this real-world national database is the huge difference in the number of cases treated with LHRH agonists versus degarelix. In the study of Davey et al. (23) on 9081 ADT observations, only 101 were under degarelix treatment, and also in the other trials, more than 90% of cases were treated with an LHRH agonist. Moreover, it is not clear whether the two populations (LHRH agonists versus degarelix) were well balanced in terms of baseline risk factors for CV events and how PC stages (metastatic versus non-metastatic) were distributed.

Again, considering the limited follow-up of observation, also in this real-world analysis it is not possible to establish the different long-term effects of LHRH agonists versus degarelix in terms of CV events.

Unfortunately, all these points can strongly condition the differences in CVD rates observed in this real-world analysis. Finally, real-life data not homogeneously sustain a lower risk for CV events using degarelix versus LHRH agonists and the contrasting results reported by the studies sustain the suggestion that differences are strongly conditioned by significant limitations in terms of populations (**Table 3**).

**Table 3:** Clinical perspectives.

Clinical point	Level of evidence
The age-related decline in testosterone serum levels in men is a possible cause for the increased risk of hypertension and cardiovascular diseases	**strong**
Prostate cancer survivors have a higher risk of developing or dying from CVD compared to the general population	**strong**
The long-term use of ADT is related to a significant increase in the risk of CVD and CVI	**week**
In the first 12 months of treatment degarelix is associated to a lower incidence of CVD when compared to LHRH agonists. This evidence is not homogeneously confirmed in real-world analysis and in follow-up longer than 1 year	**strong**
In PC cases with pre-existing CVDs, in the first 12 months of treatment degarelix is associated with a lower risk of new CV events	**week**

(ADT, androgen deprivation therapy; CVD, cardiovascular disease; CVI, cardiovascular intervention).

Moreover, considering that guidelines recommend in metastatic hormone-sensitive PC (mHSPC) to combine ADT with new strategies such as docetaxel or new generation androgen target therapies (enzalutamide, abiraterone, apalutamide), an analysis on the CV safety related only to monotherapies using GnRH antagonist versus LHRH agonists could be no more useful.

Few unfit mHSPC patients will be still treated with traditional ADT alone and therefore a more actual topic is whether the different combination of degarelix versus LHRH agonists with new generation hormone therapies can condition CV events. This analysis cannot be obtained by the randomized trials that determined recommendations for these new therapeutic indications in mHSPC, where mainly all cases were submitted as standard ADT to LHRH agonists. Real-world analysis on more balanced populations involving new hormone strategies and using either degarelix or LHRH agonists as standard ADT are waited.

## Conclusions

The age-related decline in testosterone levels may represent a factor related to the increase in CVD in males. However, the potential role of ADT through castration in increasing CV events remains controversial. Randomized trials sustain a possible protective role for degarelix versus LHRH agonist in reducing the rate of new CV events and interventions in the short-term period (12 months). The strength of evidence is limited by the study’s design that excludes an evaluation longer than 1 year and does not correctly stratify populations. Real-word data are contradictory in different national experiences and are strongly condition by huge differences between the two groups of treatment. This kind of analysis should be extended to the new combination strategies recommended by guidelines in mHSPC.

## Author Contributions

AS conceived and designed the study and performed the literature search. AS and GMB drafted the manuscript. FC participated in editing and revisons of the manuscript. GC supervised the study and made the critical revision. SS, FDG, MM, MF, EDB, RMS, FP, LC, RD, WR contributed to the article and approved the submitted version

## Funding

This paper has been published with the financial support of Ferring. The authors declare that this study received funding from Ferring. The funder was not involved in the study design, collection, analysis, interpretation of data, the writing of this article or the decision to submit it for publication.

## Conflict of Interest

The authors declare that the research was conducted in the absence of any commercial or financial relationships that could be construed as a potential conflict of interest.
